# Potential of HIV Self-Sampling to Increase Testing Frequency Among Gay, Bisexual, and Other Men Who Have Sex With Men, and the Role of Online Result Communication: Online Cross-Sectional Study

**DOI:** 10.2196/21268

**Published:** 2020-11-30

**Authors:** Tomás Maté, Juan Hoyos, Juan Miguel Guerras, Cristina Agustí, Sophocles Chanos, Matthias Kuske, Ricardo Fuertes, Roxana Stefanescu, Jose Pulido, Luis Sordo, Luis de la Fuente, María José Belza, EURO HIV EDAT Group

**Affiliations:** 1 Gerencia Regional de Salud de Castilla y León Valladolid Spain; 2 CIBER Epidemiología y Salud Pública (CIBERESP) Madrid Spain; 3 Departamento de Salud Pública y Materno-Infantil Universidad Complutense de Madrid Madrid Spain; 4 Centro Nacional de Epidemiología Instituto de Salud Carlos III Madrid Spain; 5 Departament de Salut Centre Estudis Epidemiologics sobre les Infeccions de Transmissio Sexual i Sida de Catalunya (CEEISCAT) Generalitat de Catalunya Badalona Spain; 6 Checkpoint Athens Athens Greece; 7 AIDS Hilfe NRW e.V. Berlin Germany; 8 GAT-Grupo de Ativistas em Tratamentos Lisboa Portugal; 9 ARAS-Asociatia Romana Anti-SIDA Bucharest Romania; 10 Escuela Nacional de Sanidad Instituto de Salud Carlos III Madrid Spain; 11 See Acknowledgments

**Keywords:** early diagnosis, HIV, testing, men who have sex with men, online testing, MSM, diagnosis, self-sampling, frequency, cross-sectional, communication

## Abstract

**Background:**

Late HIV diagnosis remains frequent among the gay, bisexual, and other men who have sex with men (GBMSM) population across Europe. HIV self-sampling could help remove barriers and facilitate access to testing for this high-risk population.

**Objective:**

We assessed the capacity of HIV self-sampling to increase the testing frequency among GBMSM living in Denmark, Germany, Greece, Portugal, Romania, and Spain, and evaluated the role of new technologies in the result communication phase.

**Methods:**

We analyzed a convenience sample of 5019 GBMSM with prior HIV testing experience who were recruited during 2016 through gay dating websites. We estimated the proportion of GBMSM who reported that the availability of self-sampling would result in an increase of their current testing frequency. We constructed a Poisson regression model for each country to calculate prevalence ratios and 95% CIs of factors associated with an increase of testing frequency as a result of self-sampling availability.

**Results:**

Overall, 59% (between country range 54.2%-77.2%) of the participants considered that they would test more frequently for HIV if self-sampling was available in their country. In the multivariate analysis, the increase of testing frequency as a result of self-sampling availability was independently associated with reporting a higher number of unprotected anal intercourse events in all countries except for Greece. Independent associations were also observed among GBMSM who were not open about their sex life in Germany, Greece, Portugal, and Spain; those with a lower number of previous HIV tests in Denmark, Greece, Portugal, and Spain; and for those that took their last test more than 3 months previously in Germany, Portugal, Romania, and Spain. In addition, 58.4% (range 40.5%-73.6%) of the participants indicated a preference for learning their result through one-way interaction methods, mainly via email (25.6%, range 16.8%-35.2%) and through a secure website (20.3%, range 7.3%-23.7%). Almost two thirds (65%) of GBMSM indicated preferring one of these methods even if the result was reactive.

**Conclusions:**

Availability of HIV self-sampling kits as an additional testing methodology would lead to a much-needed increase of testing frequency, especially for the hidden, high-risk, and undertested GBMSM population. Online-based technologies without any personal interaction were preferred for the communication of the results, even for reactive results.

## Introduction

HIV remains a significant public health problem in Europe. In 2017, 26,614 individuals were diagnosed as having HIV in the European Union/European Economic Area. Sex between men remains the most common transmission route and accounts for half of all new diagnoses [1]. A keystone of international strategies designed to curtail the HIV epidemic is to expand testing to reduce the undiagnosed fraction of the epidemic [2,3]. Estimates in 2018 indicated that there were 776,086 and 31,427 people living with HIV in western and central Europe, 13% and 17% of whom remained undiagnosed, respectively [4]. Delayed diagnosis remains common across Europe, and 53% of all new diagnoses are made at a late stage of infection (CD4 count of <350 mm^3^). Although gay, bisexual, and other men who have sex with men (GBMSM) are less affected by a delayed diagnosis in relative terms (41.0%), they comprise the group with the highest absolute number of delayed diagnoses [1].

The promotion of HIV testing is in fact a vital point of the 90-90-90 UNAIDS plan to end the AIDS epidemic. According to this plan, by 2030, 90% of all people living with HIV should have a diagnosis, 90% of the diagnosed population should be on treatment, and 90% of these should achieve viral suppression [2]. The benefits of promoting earlier diagnosis are two-fold. From a public health perspective, testing enables early access to highly active antiretroviral therapy that leads to the reduction of viral load and onward transmission [5]. Receiving an HIV diagnosis also leads to short-term behavioral changes, and individuals tend to reduce their engagement in sexual risk behaviors [6]. From the patient perspective, early diagnosis has benefits of reducing morbidity and mortality [7,8]. In fact, if HIV is diagnosed and treated early, the life expectancy is similar to that of the general population [8].

In light of the importance of HIV testing, several strategies have been implemented in the last decade both in clinical and nonclinical settings [9,10]. In the case of GBMSM, recommendations are that they should be tested at least every 12 months [11]; however, a large proportion of this population still does not meet the proposed testing frequency in Europe [12]. Thus, there is still room for improvement. One of the strategies that has been rolled out to promote earlier diagnosis is HIV self-sampling, also known as home sampling or postal sampling. In this testing methodology, an individual collects his/her own blood or saliva sample using a suitable kit. The sample will then be posted to a designated laboratory for processing. Results are then delivered by phone, text message, online, or face to face.

With the exception of examples in the United Kingdom [13], Belgium [14,15], and Spain [16], this strategy has rarely been studied in Europe [17], and its capacity for increasing the HIV testing frequency among GBMSM remains unknown. Additionally, a key element that needs to be factored in when considering the introduction of self-sampling as an additional testing strategy is the preference regarding methods of result communication and if it would change depending on the result received. This aspect has never been studied and would help to shape the design of this testing strategy to the different national scenarios so as to achieve optimal linkage to HIV care.

In this study, we analyzed how self-sampling would contribute to the increase of testing frequency among GBMSM who were recruited online in 6 European countries where this testing option is yet to be introduced at a national scale. Additionally, we assessed the preferred methods of communicating test results taking into account the reactiveness of the self-tested sample.

## Methods

### Design

In the context of the EURO HIV EDAT project (Operational Knowledge to Improve HIV Early Diagnosis and Treatment Among Vulnerable Groups in Europe; Grant Agreement number 2013 11 01), we conducted an online cross-sectional study in 8 European countries (Belgium, Denmark, Greece, Germany, Portugal, Romania, Slovenia, and Spain) between April and December 2016. The working team included members of academia and local organizations that ran community-based voluntary counseling and testing sites. Further information on this project can be found elsewhere [18]. The project received approval of the Ethical Committee of Investigation and Animal Welfare of the Instituto de Salud Carlos III (CEI PI 52_2015-v2) and Hospital Germans Trias i Pujol (PI-14-106).

### Recruitment Procedures

We aimed at recruiting a large convenience sample of GBMSM. The dissemination of an open survey was largely performed through GBMSM geospatial dating apps and websites that were previously identified by the local community-based voluntary counseling and testing organizations in each participating country. Likewise, we made an effort to disseminate the survey in websites of GBMSM-oriented associations and supportive organizations. Advertisement was performed through banners, direct messages, and mailing lists. Those who clicked on the promotional banner or link were directed to an introductory screen that included brief information on the aims of the project, its anonymity, funding, and the partners involved. It also included a link to the EURO HIV EDAT project website for those interested in more information. To ensure complete anonymity, no cookies or internet protocols were collected. Participation was voluntary and those who decided to go ahead gave their informed consent by checking a box with the message “I have read and understood the above information, in the country I live in I am old enough to legally have sex, and I want to participate” before moving on to the first question. No incentives were offered for participation.

### Inclusion and Exclusion Criteria

A total of 5799 HIV-negative GBMSM who resided in one of the participating countries accessed the questionnaire. We excluded 496 participants who had missing data on the question assessing our main outcome (potential of self-sampling to increase current testing frequency). Thus, the response rate was 91.45% (5303/5799). Additionally, we had to exclude participants from Slovenia (n=175) and Belgium (n=109) since the chosen dissemination websites in these two countries were not appropriately efficient, and the sample size was insufficient to run the multivariate analysis. Our final sample therefore comprised 5019 GBMSM from 6 countries.

### Data Collection Instrument

Data were collected through a self-administered online questionnaire. The questionnaire was designed in English and translated to the language of each of the analyzed countries: Danish, German, Greek, Portuguese, Romanian, and Spanish. The translation was carried out by the partners of each participating country, all of whom were native speakers. Upon completion, questionnaires were back-translated using Google Translate to check for mistakes and inconsistencies with the English version. 

The questionnaire included 90 items (1 item per page) although not every participant had to answer all questions. The completion time was approximately 20 minutes, which included sections to assess sociodemography, HIV testing history, outness about sex life with other men, sexual risk behaviors, and past diagnosis of sexually transmitted infections. To assess outness, we asked the participants “How would you describe the way you live your sex life with men?” This question had four response options: “openly,” “discreetly,” “hidden,” “in total secrecy.” The questionnaire also included a section that assessed several aspects related to HIV self-sampling. Before starting this section, the following definition was presented to participants: “Regarding self-sampling, sometimes referred to as ‘Postal Tests,’ you use a ‘kit’ to take either a blood or a saliva sample. This will then be posted to a laboratory for analysis and the results will be fed back to you.” Our main outcome was the potential of self-sampling to increase current testing frequency, which was assessed through the following question: “If HIV self-sampling was to be made available, you would test…”: (1) more times/more frequently than now, (2) about the same, or (3) less than now. This question was answered only by HIV-negative GBMSM.

We also assessed the role of new technologies in the delivery of test results using the following question: “When the laboratory has your results, how would you like to receive them?” Participants could choose one of the following answers: (1) by email, (2) through a secure website, (3) By SMS text messaging, (4) through a face-to-face consultation at a medical office, (5) through a face-to-face consultation at a community-based organization or a nongovernmental organization, (6) by phone call, and (7) other. Those who chose options 1 to 3 were also asked: “What if the result was reactive, how would you prefer to receive it then?” They then had to choose between the following options: (1) I would choose the same option, (2) I would rather receive a phone call, (3) I would rather attend a face-to-face consultation at a medical office, (4) I would rather attend a face-to-face consultation at a community-based organization, and (5) other. Respondents were able to review and change their answers through use of a “Back” button. The contents of the instrument can be found in Multimedia Appendix 1. Data collection was anonymous (no questions including personal identification were included) and confidential. Before the launch, the questionnaire was piloted and revised by partners from all countries. No randomization of questionnaire items was performed.

### Data Analysis

We performed a descriptive analysis of the main characteristics of the sample by country of residence. The data for the total number of participants were weighted to adjust for the disproportionate distribution of the sample by country of recruitment. Weighting coefficients were calculated using the male population between 18 and 65 years old living in the participating countries in 2016. Data were extracted from EUROSTAT [17]. Only weighted percentages are presented for the weighted total population.

For each country, we calculated the prevalence of participants who reported that the availability of HIV self-sampling kits would result in an increase of their testing frequency by relevant sociodemographic, behavioral, and HIV testing history variables. To estimate the factors associated with an increase of the testing frequency due to HIV self-sampling, we conducted a Poisson regression with robust variance multivariate analysis to estimate the crude and adjusted prevalence ratios (PRs) with their 95% CIs. A multivariate model was built for each country. We chose Poisson regression with robust variance because it is a better alternative than logistic regression when working with frequent outcomes [19,20]. We initially included all of the relevant variables with a significance level ≤.20 and used the minimum Akaike information criterion and the minimum Bayesian Schwartz information criterion for model comparisons and to select the optimal model.

Finally, we assessed the preferred methods of result communication by country. We grouped methods in two groups depending on the information flow they allowed: (1) unidirectional methods, in which communication flows only toward the user with no possibility of asking questions or giving direct feedback (email, secure website, SMS text messaging); and (2) bidirectional methods, in which communication can flow two ways (medical office, community-based organizations/nongovernmental organizations, phone call).

For those who chose a unidirectional method, we estimated the proportion that would have chosen the same testing method even if the result was reactive. Data on the total number of participants were also weighted using the aforementioned method.

## Results

### Participant Characteristics

The main demographic and HIV-related characteristics are summarized in Table 1. Among the total 5019 participants, 72.4% were between 25-49 years of age and 90.5% were born in their current country of residence. Approximately one third lived in cities with under 100,000 inhabitants, ranging from 26.4% (132/499) in Greece to 46.8% (247/528) in Portugal, and 47.3% had not finished a university degree at the time of the survey, ranging from 32.8% (165/503) in Greece to 56.5% (530/938) in Germany.

Overall, 20.2% of the participants indicated that they kept their sex life with other men hidden or in total secrecy, with the lowest proportion in Denmark and the highest in Romania. Regarding sexual risk indicators, 64.2% reported having had unprotected anal intercourse in the last 12 months with at least one partner, ranging from 48% (172/358) in Greece to 76.5% (186/243) in Denmark, and 41.8% had previously been diagnosed with a sexually transmitted infection, ranging from 31.2% (60/192) in Romania to 55.2% (134/243) in Denmark.

Overall, 19.9% self-reported having been tested for HIV at least once in the past, ranging from 13.1% (42/320) in Denmark to 28% (86/307) in Romania, and 39.1% had received their last test more than 12 months previously, ranging from 27% (136/502) in Greece to 43.1% (404/938) in Germany.

**Table 1:** Sociodemographic profile, outness, sexual behaviors, history of sexually transmitted infections (STIs), and testing history of the participants.

Characteristic		Denmark (N=320), n (%)		Germany (N=941), n (%)		Greece (N=503), n (%)		Portugal (N=531), n (%)		Romania (N=307), n (%)		Spain (N=2417), n (%)		Total^a^ (N=5019)	
**Age (years)**															
	<25	35 (10.9)		57 (6.1)		79 (15.7)		46 (8.7)		44 (14.3)		255 (10.6)		9.1	
	25-29	48 (15.0)		108 (11.5)		78 (15.5)		66 (12.4)		59 (19.2)		395 (16.3)		14.1	
	30-34	41 (12.8)		132 (14.0)		81 (16.1)		84 (15.8)		64 (20.8)		388 (16.1)		15.5	
	35-39	54 (16.9)		149 (15.8)		111 (22.1)		95 (17.9)		45 (14.7)		364 (15.1)		16.0	
	40-49	93 (29.1)		276 (29.3)		104 (20.7)		139 (26.2)		62 (20.2)		636 (26.3)		26.8	
	≥50	49 (15.3)		219 (23.3)		50 (9.9)		101 (19.0)		33 (10.7)		379 (15.7)		18.5	
**Place of birth**															
	In country of current residence	275 (86.2)		844 (90.5)		475 (97.3)		463 (87.5)		299 (98.0)		2076 (86.8)		90.5	
	Europe	26 (8.2)		58 (6.2)		7 (1.4)		17 (3.2)		4 (1.3)		100 (4.2)		4.7	
	Other	18 (5.6)		31 (3.3)		6 (1.2)		49 (9.3)		2 (0.7)		215 (9.0)		4.8	
**Number of inhabitants in place of residence**															
	≥1,000,000	111 (34.8)		260 (27.7)		269 (53.9)		104 (19.7)		87 (28.5)		865 (35.9)		31.3	
	100,000-999.999	104 (32.6)		336 (35.8)		98 (19.6)		177 (33.5)		130 (42.6)		844 (35.0)		35.2	
	10,000-99,999	66 (20.7)		233 (24.8)		98 (19.6)		169 (32.0)		58 (19.0)		515 (21.4)		23.2	
	<10,000	38 (11.9)		110 (11.7)		34 (6.8)		78 (14.8)		30 (9.8)		186 (7.7)		10.3	
**Education**															
	Upper secondary education	140 (43.9)		309 (32.9)		72 (14.3)		170 (32.1)		84 (27.7)		632 (26.2)		29.7	
	Postsecondary/nontertiary education	11 (3.4)		221 (23.6)		93 (18.5)		30 (5.7)		35 (11.6)		331 (13.7)		17.6	
	University education	168 (52.7)		408 (43.5)		338 (67.2)		330 (62.3)		184 (60.7)		1450 (60.1)		52.6	
**Lives sex life with men…**															
	Openly	244 (76.3)		498 (53.0)		94 (18.7)		107 (20.2)		38 (12.4)		1110 (45.9)		43.3	
	Discreetly	56 (17.5)		267 (28.4)		239 (47.5)		285 (53.7)		158 (51.5)		977 (40.4)		36.4	
	Hidden/In total secrecy	20 (6.3)		175 (18.6)		170 (33.8)		139 (26.2)		111 (36.2)		329 (13.6)		20.2	
**Number of partners with unprotected anal intercourse (last 12 months)**															
	None	57 (23.5)		255 (36.2)		186 (52.0)		136 (33.4)		54 (27.6)		692 (37.1)		35.8	
	1	63 (25.9)		208 (29.5)		116 (32.4)		127 (31.2)		73 (37.2)		613 (32.8)		31.4	
	2-4	66 (27.2)		153 (21.7)		43 (12.0)		100 (24.6)		49 (25.0)		372 (19.9)		21.4	
	≥5	57 (23.5)		89 (12.6)		13 (3.6)		44 (10.8)		20 (10.2)		190 (10.2)		11.4	
**History of STI diagnosis**															
	Last 12 months	34 (14.0)		70 (10.0)		41 (11.6)		59 (14.8)		15 (7.8)		203 (11.0)		10.5	
	>12 months ago	100 (41.2)		229 (32.8)		90 (25.6)		111 (27.9)		45 (23.4)		608 (32.9)		31.3	
	None	109 (44.9)		399 (57.2)		221 (62.8)		228 (57.3)		132 (68.8)		1038 (56.1)		58.2	
**Number of HIV tests (ever)**															
	1	42 (13.1)		176 (18.7)		95 (18.9)		97 (18.3)		86 (28.0)		484 (20.0)		19.9	
	2-5	122 (38.1)		447 (47.5)		239 (47.5)		243 (45.8)		154 (50.2)		1144 (47.3)		47.4	
	6-9	64 (20.0)		148 (15.7)		71 (14.1)		87 (16.4)		31 (10.1)		382 (15.8)		15.2	
	≥10	92 (28.8)		170 (18.1)		98 (19.5)		104 (19.6)		36 (11.7)		407 (16.8)		17.5	
**Time since last HIV test**															
	≤3 months	84 (26.3)		197 (21.0)		184 (36.7)		112 (21.1)		69 (22.5)		611 (25.4)		23.4	
	3-12 months	125 (39.2)		337 (35.9)		182 (36.3)		238 (44.9)		117 (38.1)		921 (38.2)		37.4	
	1-2 years	57 (17.9)		165 (17.6)		69 (13.7)		87 (16.4)		60 (19.5)		433 (18.0)		17.6	
	>2 years	53 (16.6)		239 (25.5)		67 (13.3)		93 (17.5)		61 (19.9)		445 (18.5)		21.5	

^a^ Weighted percentages, calculated exclusively using valid data.

### Factors Associated With Potential of Self-Sampling to Increase Current Testing Frequency

Regarding the potential of self-sampling for increasing the current frequency of HIV testing, 59% (54.2% in Spain, 55.9% in Germany, 59.1% in Denmark, 63.0% in Greece, 66.7% in Portugal, and 77.2% in Romania) indicated that, if available in their country of residence, they would test more frequently. Proportions of participants reporting an increase of testing frequency as a result of the availability of self-sampling by relevant variables can be found in Multimedia Appendix 2.

Table 2 shows the results of the multivariate analysis performed for each of the 6 countries separately. The variables that were independently associated with the potential of self-sampling to increase testing frequency were outness about sex life with other men, number of partners with whom they had unprotected anal intercourse in the last 12 months, number of lifetime HIV tests performed, and time since their last HIV test.

With the exception of GBMSM living in Denmark and Romania, the potential of self-sampling to increase testing frequency was higher among those who lived their sex life discreetly or “hidden/in total secrecy” than among participants who were open about their sex lives.

The number of partners with whom participants reported having engaged in unprotected anal intercourse was also associated with the capacity of self-sampling to increase testing frequency in all countries except for Greece. We found significant adjusted PRs among Spain-based respondents who reported having engaged in unprotected anal intercourse with only one partner. In all countries, the adjusted PRs were statistically significant among those who reported being involved in unprotected anal intercourse with 2-4 partners and the PRs increased further among those who reported having had ≥5 partners in the last 12 months. The adjusted PR decreased with the lifetime number of HIV tests. This pattern was the same in all countries except for Germany and Romania, in which no statistical significance was found.

Finally, apart from Denmark and Greece, the potential of self-sampling to increase testing frequency was independently associated with time elapsed since the last HIV test. Compared to those tested ≤3 months ago, the adjusted PR gradually increased among GBMSM who were last tested between 3 and 12 months prior and peaked among those tested >12 months previously.

**Table 2:** Factors associated with a potential increase of testing frequency if self-sampling was made available.

Variable		Denmark (N=320), PR^a^ (95% CI)	Germany (N=941), PR (95% CI)	Greece (N=503), PR (95% CI)	Portugal (N=531), PR (95% CI)	Romania (N=307), PR (95% CI)	Spain (N=2417), PR (95% CI)
**Age (years)**							
	≥50	1.0 (0.7-1.4)	1.0 (0.8-1.3)	1.2 (1.0-1.5)	1.1 (0.8-1.4)	1.0 (0.8-1.2)	1.1 (0.9-1.3)
	25-49	1.0 (0.8-1.3)	1.1 (0.8-1.4)	1.1 (0.9-1.3)	1.3 (1.0-1.6)	1.0 (0.9-1.2)	1.2 (1.0-1.3)
	<25	1.0	1.0	1.0	1.0	1.0	1.0
**Education**							
	No university education	1.3 (1.1-1.5)	—^b^	—	—	—	—
	University education	1.0	—	—	—	—	—
**Lives sex life with men…**							
	Hidden/Total secrecy	—	1.4 (1.2-1.6)	1.4 (1.1-1.8)	1.2 (1.0-1.5)	—	1.3 (1.1-1.4)
	Discreetly	—	1.3 (1.1-1.4)	1.2 (0.9-1.5)	1.0 (0.9-1.3)	—	1.2 (1.1-1.3)
	Openly	—	1.0	1.0	1.0	—	1.0
**Number of partners with unprotected anal intercourse (last 12 months)**							
	≥5	1.5 (1.1-2.1)	1.3 (1.0-1.6)	—	1.3 (1.1-1.7)	1.3 (1.1-1.6)	1.5 (1.3-1.7)
	2-4	1.4 (1.0-2.0)	1.3 (1.1-1.5)	—	1.3 (1.1-1.5)	1.3 (1.1-1.6)	1.3 (1.2-1.5)
	1	1.1 (0.8-1.6)	1.1 (0.9-1.3)	—	1.1 (0.9-1.4)	1.1 (0.9-1.3)	1.1 (1.0-1.3)
	None	1.0	1.0	—	1.0	1.0	1.0
**Number of HIV tests (ever)**							
	1	1.5 (1.2-2.0)	—	1.7 (1.3-2.2)	1.7 (1.3-2.1)	—	1.5 (1.3-1.8)
	2-5	1.3 (1.0-1.7)	—	1.7 (1.3-2.1)	1.5 (1.2-2.0)	—	1.4 (1.2-1.6)
	6-9	1.2 (0.9-1.6)	—	1.3 (0.9-1.8)	1.3 (1.0-1.8)	—	1.1 (1.0-1.3)
	≥10	1.0	—	1.0	1.0	—	1.0
**Time since last HIV test**							
	>12 months	—	1.3 (1.1-1.6)	—	1.4 (1.1-1.8)	1.4 (1.1-1.7)	1.3 (1.1-1.4)
	3-12 months	—	1.1 (0.9-1.3)	—	1.3 (1.1-1.6)	1.4 (1.1-1.7)	1.1 (1.0-1.3)
	≤3 months	—	1.0	—	1.0	1.0	1.0

^a^PR: adjusted prevalence ratio.

^b^Variable not included in multivariate analysis.

### Preferences Regarding Method of Test Result Communication

Table 3 presents the results of the participants’ preferences regarding the method of result communication. The majority of respondents preferred unidirectional methods of communication (email, secure website, and SMS text messaging). The preferred bidirectional method was face-to-face consultation at a medical office and the least preferred method was a phone call. Nearly two-thirds of those who chose unidirectional methods indicated that they would retain this preference over other methods even if the self-sampling result was determined to be reactive.

**Table 3:** Preferred HIV test result communication method in general and for a reactive result.

Communication Method			Denmark (N=320), n (%)	Germany (N=941), n (%)	Greece (N=503), n (%)	Portugal (N=531), n (%)	Romania (N=307), n (%)	Spain (N=2417), n (%)	Total^a^ (N=5019)
**Unidirectional**									
	All		151 (52.8)	467 (53.6)	195 (40.5)	377 (73.6)	187 (62.5)	1518 (66.4)	58.4
	**Email**								
		Overall	48 (16.8)	179 (20.6)	88 (18.3)	180 (35.2)	96 (32.1)	738 (32.3)	25.6
		Same response if result is reactive	30 (62.5)	126 (70.4)	52 (59.1)	120 (66.7)	61 (63.5)	437 (59.2)	65.7
	**Secure website**								
		Overall	48 (16.8)	206 (23.7)	35 (7.3)	109 (21.3)	46 (15.4)	445 (19.5)	20.3
		Same response if result is reactive	35 (72.9)	144 (69.9)	20 (57.1)	73 (67.0)	29 (63.0)	254 (57.1)	65.0
	**SMS text message**								
		Overall	55 (19.2)	82 (9.4)	72 (14.9)	88 (17.2)	45 (15.1)	335 (14.7)	12.5
		Same response if test is reactive	33 (60.0)	53 (64.6)	38 (52.8)	57 (64.8)	30 (66.7)	186 (55.5)	62.1
**Bidirectional**									
	All		132 (46.2)	371 (42.6)	282 (58.5)	131 (25.6)	105 (35.1)	729 (31.9)	39.0
	Medical office		55 (19.2)	220 (25.3)	139 (28.8)	66 (12.9)	75 (25.1)	522 (22.8)	23.9
	At a CBO^b^/NGO^c^		33 (11.5)	90 (10.3)	82 (17.0)	52 (10.2)	10 (3.3)	80 (3.5)	8.1
	Phone call		44 (15.4)	61 (7.0)	61 (12.7)	13 (2.5)	20 (6.7)	127 (5.6)	6.9
Other			3 (1.0)	33 (3.8)	5 (1.0)	4 (0.8)	7 (2.3)	39 (1.7)	2.7

^a^Weighted percentages, calculated exclusively using valid data.

^b^CBO: community-based organization.

^c^NGO: nongovernmental organization.

## Discussion

Our study shows that HIV self-sampling has high potential to increase the frequency of HIV testing among GBMSM in the 6 European countries studied. More than half of the respondents reported that, if made available, self-sampling would increase their testing frequency. This potential was found to be especially high among those who are not open about their sexuality, who had a greater number of sexual partners, and for those who are undertested. Based on the preferences expressed by the participants, online technologies should play a key role in communicating test results.

The evidence base on HIV self-sampling kits is still very weak. Two studies from Belgium [14,15] and one study from Spain [16] have proven that self-sampling kits can be used in outreach activities at locations frequented by populations at high risk, including GBMSM [14,16]. In the United Kingdom, the national HIV self-sampling service, which is fully online-based, has been running since 2015 [13]. Outside of these three countries, the only European study we have found that assessed this diagnostic strategy was also based on the EURO HIV EDAT project and concluded that self-sampling, although not very well known, had high potential of use in European countries [17]. This is the first study to address the question as to whether the introduction of this method would result in an increase of testing frequency. Our data suggest that the availability of HIV-self sampling kits would lead to increased testing rates in several key subpopulations.

Subpopulations who do not live their sex life openly are hard to reach and are the least likely to encounter or access health promotion interventions [12]. Thus, GBMSM who are the least open about their sex life generally presented lower testing rates in the last 12 months than the rest of the GBMSM [12]. Some characteristics of self-sampling methodologies such as privacy and not having to reveal sexual orientation or discuss their sex life with a clinician could facilitate testing for GBMSM that have yet to come to terms with their own sexuality. Based on our data, this hypothesis is plausible since the reported potential of self-sampling kits to increase testing frequency was especially high among GBMSM who are not completely open about their sex lives.

The ultimate goal of the introduction of strategies such as self-sampling is to promote testing and facilitate testing to undertested populations. Current testing recommendations for GBMSM are demanding, and barriers such as fear of stigma and discrimination, lack of anonymity or confidentiality, and waiting times at testing sites [21] have been described as deterrents to testing. Our results suggest that self-sampling could help to alleviate some of these barriers, leading to an increase of the number of tests performed and less time between testing in undertested individuals. This could mean that a fraction of those who are not meeting testing recommendations could do so if self-sampling kits are available, allowing for seroconversions to be detected earlier.

From a public health perspective, shortening the time from infection to diagnosis by increasing testing frequency is particularly relevant in populations with a high number of sexual partners. Those who reported being involved in unprotected anal intercourse with a higher number of partners presented higher probabilities of unknowingly transmitting or acquiring HIV from partners of a serodiscordant status. According to our results, the introduction of self-sampling would increase testing frequency precisely in this key population. In this study, we only considered condom use during anal intercourse and we did not assess other risk reduction strategies such as serosorting. Those reporting a higher number of unprotected anal intercourse events could be having unprotected anal sex only with partners of a presumably equal (negative) serostatus (serosorting) or with HIV-positive partners with a presumably undetectable viral load to avoid infection [22,23]. Nevertheless, the effectiveness of serosorting is questionable [24-26] and the percentage of virally suppressed HIV-positive GBMSM varies widely across countries [4]; thus, the impact of self-sampling in the testing frequency of GBMSM who use this protective strategy needs to be better understood.

Additionally, we did not assess HIV pre-exposure prophylaxis (PREP). Those involved in unprotected anal intercourse could be receiving PREP but this is unlikely since it was not widely available at the time of data collection (2016). Nevertheless, PREP has now been incorporated into the preventive options among GBMSM, and the role that HIV self-sampling could play in the follow-up of patients on PREP merits further study. Conversely, postexposure prophylaxis is an available option and is an effective means of protection against HIV. We also did not assess if some of those involved in unprotected sex could be doing so with the intention of taking postexposure prophylaxis after the risk exposure; however, this is highly unlikely since uptake is very low among GBMSM across Europe [12].

When assessing the introduction of self-sampling, it is important to consider several steps along the testing process, namely the distribution of the testing kits and the consultation of the test result. The aforementioned examples of studies assessing self-sampling kits distributed during outreach activities in locations frequented by GBMSM, such as saunas or gay venues, were able to conduct a modest number of tests [14-16]. Consequently, the number of new HIV diagnoses was also limited. Another approach is to embed the distribution of self-sampling kits in already existing health services and community organizations. There is a gap of knowledge regarding the acceptability of this approach for GBMSM, but an assessment focusing on black Africans of all sexualities concluded that, for several reasons, the number of self-sampling kits distributed and conducted based on this distribution pathway was also very low [27]. In this sense, methodologies fully based on internet distribution have proven to be by far the most efficient. In the United Kingdom, this methodology has facilitated testing for more than 69,000 self-samples, 788 of which were found to be reactive [13]. When introducing HIV self-sampling in national testing policies, countries need to first consider online distribution. This is particularly essential in the case of GBMSM considering that online gay dating apps and websites offer an extremely efficient platform to reach out to individuals for conducting public health interventions.

Timely linkage to care following an HIV diagnosis is also critical since late access can result in worse patient outcomes [28]. This is particularly relevant when considering testing methodologies outside of clinical settings such as that assessed in this study; thus, countries need to establish robust confirmation routes to assure optimal linkage to care. In self-sampling methodologies, result consultation needs to be taken into account. With the exception of Greece, internet-based methods were preferred by our participants, even in the case of receiving a reactive result. These preferences need to be considered since it is of utter importance that tested individuals obtain further consultation of their results, especially if reactive, as this is the first step for result confirmation and consequent linkage to care. The use of digital communication technologies has proven to be effective in promoting testing among GBMSM [29]; however, studies are needed to assess if they are also capable of providing timely confirmation and linkage to care for those using self-sampling methodologies.

Our results are not without limitations. Our sample was mainly recruited via gay dating apps and websites. Although these platforms are increasingly used by GBMSM as a way of socializing and meeting new sexual partners, generalization of these results to the overall GBMSM population needs to be made with caution, especially given the limited sample size in some countries. In this sense, GBMSM identifying as gay and reporting more sexual risk behaviors could be overrepresented in our sample as has been previously reported [30]. Another limitation of this study is that we are working on a hypothetical situation. Although there is some evidence suggesting that answers based on hypothetical situations are able to predict actual behaviors [31-33], it is unclear if this is also the case with respect to the capacity of self-sampling to increase testing frequency. The definition of self-sampling included in the questionnaire explicitly mentioned the two types of self-sampling kits: saliva and blood-based kits. Thus, participants likely gave their answer considering both samples as potential options. This should also be taken into account when interpreting our results. For example, it is possible that if only one of the two sample options was made available, the proportion of participants reporting an increase of testing frequency could have been lower. Price (if marketed) and implementation problems resulting in invalid self-samples or delays in the communication of results could affect the capacity of HIV self-sampling to increase testing frequency. These barriers have not been assessed and merit further study.

As a way of ensuring complete anonymity and confidentiality, we did not collect internet protocols or cookies, and therefore could not assess the possibility of an individual answering the questionnaire more than once. However, the overall objective of the survey was clearly explained in the access screen, and given that no compensation was provided in exchange for participation, this situation was highly unlikely. An important strength is that the assessment of HIV self-sampling was performed in a set of countries that had not introduced this testing option at the time of conducting the survey. This provides baseline information to policymakers that could guide them in the introduction of this methodology.

Based on our results, national HIV testing policies in the 6 countries evaluated in this study should consider the incorporation of HIV self-sampling as an additional testing option, since its introduction could increase the testing frequency in a high proportion of GBMSM, especially among those who are not open about their sex lives, who remain undertested, and who report a high frequency of high-risk sexual behaviors. When designing its implementation, priority needs to be given to online methods of result communication, accompanied by clear referral pathways to HIV care for those obtaining a reactive result.

## Appendix

Multimedia Appendix 1 Questionnaire.

Multimedia Appendix 2 Proportion of participants who reported that HIV self-sampling would increase their testing frequency by relevant variables.

## Abbreviations

GBMSM: gay, bisexual, and other men who have sex with men
PR: prevalence ratio
PREP: pre-exposure prophylaxis
