# Supplementary material for: Potential of HIV Self-Sampling to Increase Testing Frequency Among Gay, Bisexual, and Other Men Who Have Sex With Men, and the Role of Online Result Communication: Online Cross-Sectional Study
Source: J Med Internet Res. 2020 Nov 30;22(11):e21268. doi: 10.2196/21268 (PMC7735895; doi:10.2196/21268)
Supplement: Multimedia Appendix 1 [file jmir_v22i11e21268_app1.docx]

**INTRODUCTION**

1. In what language do you want to complete this questionnaire?
   1. Danish
   2. Dutch

2. English

3. French

4. German

5. Portuguese

6. Romanian

7. Slovenian

8. Spanish

This survey focuses on Men who have sex with men. We will ask you about experiences and opinions on several innovative testing options aimed at promoting earlier HIV diagnosis including self-sampling, self-testing, rapid testing in non-conventional settings.

It is completely **ANONYMOUS**: we do NOT collect IP addresses or install any cookies, nor collect any information that can allow anybody to identify you.

We are an international group of researchers and health workers from public health institutes and non-governmental organizations from 7 European countries. This survey is funded by the European Commission (Grant Number 20131101).

For further information please visit [www.eurohivedat.](http://www.eurohivedat.)eu

To participate please tick the next box:

I have read and understood the above information, in the country I live in I am old enough to legally have sex, and I want to participate

**ABOUT YOU**

**Q1. Are you...** [Compulsory question]

1. A man
2. A woman
3. Transgender/Transsexual

*[if Q1=2 OR 3]* **Q1b. This research is about men. You are very welcome to read and complete the rest of the survey however we will be unable to use your data. What would you like to do?**

1. Exit the survey now
2. Continue reading the survey

*[If Q1b=1]* ***Thank you for your interest in our project, the results will be available across Europe soon. You can get more information at*** [www.eurohivedat.](http://www.eurohivedat.)eu*. 🡺 [EXIT]*

**Q2. How old are you?** [Compulsory question]

[Write an answer]

**Q3. Which country do you CURRENTLY LIVE in?** [Compulsory question]

1. Belgium
2. Denmark
3. France
4. Germany
5. Portugal
6. Romania
7. Slovenia
8. Spain
9. Other

*[if Q3=9]* **Q3b. This research is about men living in the above mentioned countries. You are very welcome to read and complete the rest of the survey; however we will be unable to use your data. What would you like to do?**

1. Exit the survey now
2. Continue reading the survey

*[If Q3b=1]* ***Thank you for your interest in our project, the results will be available across Europe soon. You can get more information at*** [www.eurohivedat.](http://www.eurohivedat.)eu*. 🡺 [EXIT]*

**Q4. Where you born in this country?**

1. Yes
2. No

*[If Q4=2]* **Q5. Which country were you born in?**

[Write in answer]

**Q6. How many inhabitants are there in the place (city or village) where you live? (Approximately)**

1. a million or more people
2. 500,000-999,999 people
3. 100,000-499,999 people
4. 50,000-99,999 people
5. 10,000-49,999 people
6. less than 10,000 people

*[If Q6=4 or 5 or 6]* ***Q7.* Is there a city with over 100,000 inhabitants within 30 km?**

1. Yes
2. No

**Q8. What is your highest education qualification?**

1. No secondary qualification [ISCED 1]
2. Lower secondary or second stage of Basic education: designed to complete Basic education, usually following a more subject-oriented pattern [ISCED 2]
3. (upper) secondary education: more specialized education typically beginning at age 15 or 16 years and/or the end of compulsory education [ISCED 3]
4. Post-secondary, non-tertiary education: captures programs that straddle the Boundary between upper- and post-secondary education from an International point of view as pre-university courses of short vocational programs [ISCED 4]
5. Higher education university education: specific vocational training, first and second university degrees, Bachelor, Master degree [ISCED 5]
6. University education- Doctorate (PhD) [ISCED 6]

**In this survey, we use "sexual intercourse" or "sex" to mean ONLY where there is vaginal or anal penetration.**

**Q9. Given the above mentioned definition: With whom have you ever had sex in your life?**

1. I have never had sex with men or women
2. Only with women
3. More often with women, but at least once with a man
4. About equally often with men and with women
5. More often with men, but at least once with a woman
6. Only with men

*[If Q9=1 or 2]* ***Q9b.* This research is about men who have had sex with other men at least once. You are very welcome to read and complete the rest of the survey; however we will be unable to use your data. What would you like to do?**

1=Exit the survey now

2=Continue reading the survey

*[If Q9b=1]* ***Thank you for your interest in our project, the results will be soon. You can get more information at*** [www.eurohivedat.](http://www.eurohivedat.)eu*. 🡺 [EXIT]*

**Q10. How would you describe the way you live your sex-life with men?**

1. Openly
2. Discretely
3. Hidden
4. In total secrecy

**HIV TESTING HISTORY**

**Q10_2. Do you know where you can go if you wish to receive an HIV test?**
 1. Yes
 2. No

**Q11. Besides blood donations, how many times have you BEEN TESTED for HIV?** [Compulsory question]

1. Never

2. Once

3. Twice

4. 3 to 5 times

5. 6 to 9 times

6. 10 to 15 times

7. 16 to 20 times

8. more than 20 times

*[If Q11=1]* **Q12. What is the MAIN reason for not having been tested before?**

1. I feel very healthy

2. I think that, with my behaviours, I cannot be infected

3. I am afraid of the consequences for my health of a positive result.

4. I am afraid I might lose my job or not find others if the test is positive.

5. I do not have a residence or work permit, and I think that I might have problems to obtain one if the test is positive.

6. I have fear of rejection or discrimination.

7. I am afraid of outing regarding my sexual behaviours with men.

8. I don't want to go to my doctor / health centre.

9. I don't know where to go to get tested anonymously or where no one can recognize me.

10. Having to wait several days to know the result stresses me out.

11. I never happen to find the time needed/ It is not convenient enough/It is not easy for me to find the time needed.

12. I want to get tested in a private centre but I cannot afford it.

13. I feel discomfort answering intimate and personal questions.

14. I'm a blood donor.

15. Others [Specify].

*[If Q11 ≠1]*

**Q11_2.When you feel you have been exposed or have taken a risk for contracting HIV: How important is taking an HIV test for you?**

1. I test systematically
2. I think it is important to test for HIV, but I do not test always.
3. I think it is important to test for HIV, but I rarely do.
4. For me testing for HIV is not a priority
5. I have never been exposed to HIV.

**Q13. When did you LAST have an HIV test?**

1. 3 months or less

2. More than 3 months to 6 months

3. More than 6 months to 1 year

4. More than 1 year to 2 years

5. More than 2 years to 5 years

6. More than 5 years ago.

**Q14. Where did you go for your last HIV test?**

1. At a hospital or clinic as an in-patient

2. At an HIV/STI testing service or clinic

3. At the office of a medical specialist

4. General Practitioner/family doctor

5. Private laboratory

6. At a pharmacy (performed by a pharmacist)

7. At the emergency room of a hospital

8. At a blood bank, while donating blood

9. At a mobile unit

10. At the premises of a Community Based Organization or Non-Governmental Organization.

11. In a bar/pub, club or sauna

12. I used a home self-sampling kit

13. I self-tested

14. Elsewhere [Specify]

*[If Q14 ≠12 or 13]***Q15. Was it a Rapid Test? (A test that can detect if you are infected by using blood or oral fluid and give results in about 15 minutes)**

1. Yes

2. No

**Q16. This last HIV test was** [compulsory question]

1. Positive (I have HIV infection)

2. Negative (I did not have HIV infection at the time of the test)

3. I did not go to collect the results

*[If Q11 ≠1 AND Q11≠2 AND Q13≠1 AND Q16=2]* **Q17.** **In the last 5 years, you would say you have tested usually:**

1. Every 3 months

2. Every 6 months

3. Every year

4. Every two years

5. I just get tested whenever I feel I have to do it (after an unprotected sexual intercourse, for example)

**NEW** HIV TESTING **STRATEGIES**

In some countries, there are HIV tests available that allow individuals the opportunity to do an HIV test at home. There are two COMPLETELY DIFFERENT TYPES: self-sampling and self-testing. Let’s talk about them.

Regarding **self-sampling**, sometimes referred to as ‘Postal Tests’, you use a ‘kit’ to take either a blood or a saliva sample. This will then be posted to a laboratory for analysis and the results will be feed back to you.

**Q18. Did you know that in some countries (USA or UK) this testing option is already available?**

1. Yes

2. No

*[If Q11 ≠1 ]***Q19. Have you ever taken and posted a self-sample?**

1. Yes

2. No

*[If Q19=1]*

**Q20. How many times have you taken and posted a self-sample?**

1. Once

2. Twice

3. Three times

4. 4 or 5 times

5. More than 5 times

**Q21.When was the LAST TIME you used a self-sampling test?**

1. 3 months or less

2. More than 3 months to 6 months

3. More than 6 months to 1 year

4. More than 1 year to 2 years

5. More than 2 years ago

**Q22. The LAST TIME you used a self-sampling test, you used**

1. A blood test (by finger prick)

2. A saliva test (using a mouth swab)

**Q23: How did you receive the results?**

1. By SMS

2. By phone call

3. By email

4. Through a secure website

5. Through a face to face consultation at a medical office

6. Through a face to face consultation at a Community Based Organization or Non-Governmental Organization.

7. Others [Specify]

*[If Q16=1]* **Q24.If HIV self-sampling test had been available in your country: do you think you would have used one before you were diagnosed with HIV**

1. Yes, for sure

2. Probably yes

3. I'm not sure

4. Probably no

5. No, for sure

*[If Q16=2 OR if Q11=1]*

**If self-sampling was available in your country:**

**Q25. do you think you would have used it in the past?**

1. Yes, for sure

2. Probably yes

3. I'm not sure

4. Probably no

5. No, for sure

**Usually, there are two types of HIV self-sampling kits with slightly different characteristics that you can see below: the mouth swab saliva test and the finger prick blood test.**

| **Mouth swab ‘saliva test’** | **Finger prick ‘blood test’** |
| --- | --- |
| Detects HIV that was caught more than 14 weeks ago | Detects HIV that was caught more than 4 weeks ago |
| This test uses a mouth swab. You have to place an absorbent pad against you gum for 2 minutes | The test uses a few drops of blood. Prick your finger using the supplied lancet. The drops are collected in a small collection tube or in a blotting paper. |
| You shouldn’t eat or drink for 30 minutes before using the mouth swab | It is not affected by the last time you ate or drank. |
| If the mouth swab reacts, there is about a 90% chance that you have HIV. | If reacts, there is over a 95% chance that you have HIV |

**Q26. Taking into consideration the above mentioned characteristics, if available in your country, which one would you, prefer to use?**

1. I would use prefer blood test

2. Even if wouldn't feel completely at ease, I would use blood

3. I would prefer a saliva test

4. I would never use this testing option

*[If Q26≠4]*

**Q27: When the laboratory has your results, how would you like to receive them?**

1. By text message

2. By phone call

3. By email

4. Through a secure website

5. Through a face to face consultation at a medical office

6. Through a face to face consultation at a Community Based Organization or Non-Governmental Organization.

7. Others [Specify]

*[If Q27=1 or 2 or 3 or 4]* **Q28. What if the result was positive, how would you prefer to receive it then?**

1. I would choose the same option

2. I would rather receive a phone call

3. I would rather attend a face to face consultation at a medical office

4. I would rather attend a face to face consultation at a Community Based Organization or Non-Governmental Organization.

5. Other ____________

[If *Q16 =2]* **Q29. If HIV self-sampling was to be made available, you would test…**

1. More times / more frequently than now.

2. About the same.

3. Less than now.

Let's talk now about HIV **self-test**: individuals take a sample, test themselves and obtain their result in under 20 minutes. Just as the home-sampling kits, they can be blood or saliva based. There is no need of sending the sample to a laboratory as they are read and interpreted by the person using it.

**Q44. Did you know that in some countries (USA, UK and France), it is possible to legally buy HIV self-testing kits without prescription??**

1. No, I had no clue

2. Yes, I knew.

[If Q44=1 🡪 Q46]

*[If Q44=2]* **Q45. How did you learn about it?**

1. Through a friend

2. Through a sexual partner

3. Through a gay association

4. Through an HIV/AIDS Association

5. I saw it advertised in a dating site

6. Through general media

7. Through gay oriented media

8. I saw an ad in a search engine

9. Through an informative website (NAM, aids Map, etc.)

10. Others [Specify]

*[If Q11 ≠1 ]* **Q30. Have you ever performed an HIV self-test? (Collect the specimen, perform the test and read the result)**

1. Yes

2. No

***[If Q30=1]***

**Q31. How many times have you used a self-test in your lifetime?**

1. Once

2. Twice

3. Three times

4. 4 or 5 times

5. More than 5 times

**Q32. When was the LAST TIME that you self-tested for HIV?**

1. 3 months or less

2. More than 3 months to 6 months

3. More than 6 months to 1 year

4. More than 1 year to 2 years

5. More than 2 years to 5 years

6. More than 5 years ago.

**Q33. How did you get this LAST HIV self-test?**

1. I bought it in a country where it is legally sold (online, at a pharmacy, drug store supermarket etc.)

2. I purchased it online

3. I took home a rapid test from my job

4. Someone I knew (friends/family etc) gave me a rapid test

5. Others [Specify]

**Q34. This LAST TIME you used**

1. A Blood HIV self-test

2. A Saliva HIV-test

3. Other [Specify]

**Q35. And, the result of that HIV self- test was**

1. Negative (non-reactive)

2. Positive (reactive)

3. Invalid

4. I couldn't understand the result

**Q36. And you used the self-test**

1. Alone by yourself

2. With someone

*[If Q36=2]* **Q37. Who was(were) the person(s) accompanying you?**

1. My steady partner

2. A non-steady partner

3. A friend

4. A family member

5. A member of a CBO

6. Medical staff

7. Others [Specify]

*[IF Q35≠1]***Q38. Did you self-test again just to double check the result was right?**

1. Yes

2. No

*[If Q38=1]* **Q39. And that second test was:**

1. Negative (non-reactive)

2. Positive (reactive)

3. I couldn't understand the result

4. Invalid

**Q40. After that, did you go for a conventional test to confirm the result? (A conventional test requires al least two visits: one to have your blood drawn and the second to receive the results)**

1. Yes

2. No

*[If Q40=1]*

**Q41 How many days did it take to you to go for getting that conventional test?**

/__/__/__/ days.

**Q42. The result was:**

1. Positive

2. Negative

3. Not clear/Unclear (and I was told to repeat the test again in some days)

4. I did not go to collect the results

*[If Q42=3]* **Q43. And that test was**

1. Positive

2. Negative

3. I decided not to go to get tested

4. I did not go to collect the results

**Q46. Which of the following statements best describes your opinion about the use of an HIV self-test without the need of a prescription?**

1. I support /I am in favour of it

2. I am against it

3. I'm not sure/I don’t have a clear opinion regarding this issue

*[If Q46=2 or 3]* **Q47. In your opinion, which is the most important reason for you to be AGAINST self-testing?**

1. Obtaining the sample, performing the test and interpreting the results should be done by a trained professional.

2. The presence of an expert to provide counselling and inform about the result is essential/indispensable.

3. I don't trust the quality of the test, so I have concerns about the validity of the results

4. Self-testing may help to maintain HIV as a matter of taboo/shame.

5. You can be forced to self-test in front of your partner.

6. Others [Specify]

*[If Q46=1 or 3]* **Q48.** **Which of the following options best describes the most important reason for you to SUPPORT self-testing?**

1. It helps to keep my privacy because no one would know

2. It helps to avoid intimate and personal questions

3. You can test whenever you want

4. It saves time, paperwork, queues, waiting time, etc.

5. It contributes to take responsibility for your health

6. It allows me to avoid the counselling.

7. It saves me from judgemental attitudes (regarding my practices, my sexual orientation…)

8. Others [Specify]

**Q49. Would you pay about 25-30 Euros for a self-test?**

1. No, never

2. No, unless I be in great distress.

3. Yes

*[IfQ49=1 OR 2]***Q50 Why?**

1. I can get a test for free

2. I can't afford that price

3. The Health System should bear with the costs

4. Other, specify:

**Q51. What would be the maximum price you would be willing to pay?**

[insert]

**Q52. Besides pharmacies, where would you like to be able to buy an HIV self-test?**

1. At supermarkets / drugstores

2. At Community Based Organization or Non-Governmental Organization.

3. Through the internet

4. Purchasing it by phone

5. At vending machines

6. I think it should be sold only in pharmacies

7. Other [specify]

**Q53. Providing information about self-testing by Community Based Organization or Non-Governmental Organization working in the field of HIV and/or with vulnerable populations is, in your opinion:**

1. Very important

2. Important

3. It is indifferent

4. It is not important to me

5. It has no relevance at all

*[If Q16=1]* **Q54. If the self-test had been available in your country, do you think you would have ever used one before you were diagnosed with HIV**

1. Yes, for sure

2. Probably yes

3. I'm not sure

4. Probably no

5. No, for sure

*[If Q16=2 OR if Q11=1]*

**If the self test was available in your country:**

**Q55. do you think you would have used it sometime in the past?**

1. Yes, for sure

2. Probably yes

3. I'm not sure

4. Probably no

5. No, for sure

*[If Q55=1 OR 2 OR 3]***Why would you use it?**

1. With self-testing, I can obtain a result quickly.
2. It gives me autonomy, I do not need to go to the doctor, laboratory or to a sexual health clinic.
3. I can offer an HIV test to my sexual partners.
4. It is anonymous and discreet
5. It is practical, I can do the whole process at my home.
6. I allows me to test more regularly.
7. It is less stressful
8. Others.Please describe

**If you have chosen more than one reason, please write down the number of the most important one for you:**

*[If Q55=3 OR 4 OR 5]* **Why would you not use it?**

1. I do not need to get tested
2. I get tested regularly at my doctors or in a sexual health clinic and I think it is ok like this
3. I think self-testing is not reliable
4. I am afraid of not using it properly
5. I do not l to know my result alone at home
6. Others.Please describe

**If you have chosen more than one reason, please write down the number of the most important one for you:**

*[If Q55=1 or 2]* **Q56. How many times do you think you would have used it?**

1. Just once

2. 2 or 3 times

3. 4-5 times

4. more than 5 times

**Q57. If at a self-test you had a positive or indeterminate result, where would you prefer to go for help and confirmation?**

1. At a hospital or clinic

2. At an HIV/STI testing service or clinic

3. At the office of a medical specialist

4. General Practitioner/family doctor

5. Private laboratory

6. At a pharmacy (where the test is performed by a pharmacist)

7. At the emergency room of a hospital

8. At a mobile unit

9. At a Community Based Organization or Non-Governmental Organization premises

10. In a bar/pub, club or sauna

11. Elsewhere [Specify]

**Q58. Consider whether it is likely that you propose a partner to self-test together in the following situations. Rate each answer from 0 (not likely) to 5 (very likely).**

| A casual partner before having condomless sex | *0* | *1* | *2* | *3* | *4* | *5* |
| --- | --- | --- | --- | --- | --- | --- |
| A casual partner, before the first sexual encounter, even if you are going to use condoms | *0* | *1* | *2* | *3* | *4* | *5* |
| A steady partner, to stop using condoms. | *0* | *1* | *2* | *3* | *4* | *5* |
| A steady partner before having sex again if you suspect/know that he has had a recent intercourse with another person. | *0* | *1* | *2* | *3* | *4* | *5* |
| A casual partner, to proof that I am or that I am not infected | *0* | *1* | *2* | *3* | *4* | *5* |
| I wouldn’t self-test with a partner | *0* | *1* | *2* | *3* | *4* | *5* |

A **Rapid Test for HIV** is a test that can detect if you are infected by using blood or oral fluid and give results in less than 20 minutes.

*[If Q16=2]* **Q59. Have you ever been tested for HIV using a rapid test?**

1. Yes

2. No

*[If 59=1]*

**Q60. When did you get tested for HIV using a rapid test for the last time?**

1. 3 months or less

2. More than 3 months to 6 months

3. More than 6 months to 1 year

4. More than 1 year to 2 years

5. More than 2 years to 5 years

6. More than 5 years ago.

**Q61. Where did you go for your last RAPID HIV test?**

1. At a hospital or clinic

2. At an HIV/STI testing service or clinic

3. At the office of a medical specialist

4. General Practitioner/family doctor

5. Private laboratory

6. At a pharmacy (where the test is performed by a pharmacist)

7. At the emergency room of a hospital

8. At a mobile unit

9. At a Community Based Organization or Non-Governmental Organization Premises

10. In a bar/pub, club or sauna

11. Elsewhere [Specify]

**In your opinion, undergoing rapid testing for HIV has made you more or less likely to:**

**Q62. Have another Rapid HIV test in the future**

1. Much less likely

2. Less likely

3. Neither more nor less likely

4. More likely

5. Much more likely

**Q63. Test for HIV more frequently**

1. Much less likely

2. Less likely

3. Neither more nor less likely

4. More likely

5. Much more likely

*[If Q16=2 OR Q11=1]]* **Q64. In general, where would you prefer to be tested for HIV using a Rapid test?**

1. At a hospital or clinic

2. At an HIV/STI testing service or clinic

3. At the office of a medical specialist

4. General Practitioner/family doctor

5. Private laboratory

6. At a pharmacy (where the test is performed by a pharmacist)

7. At the emergency room of a hospital

8. At a mobile unit

9. At a Community Based Organization or Non-Governmental Organization Premises

10. In a bar/pub, club or sauna

11. Elsewhere [Specify]

**PREFERENCES AND PATTERNS**

*[If Q11=1 or Q16=2]*

Unlike the rapid tests, the conventional testing requires a sample of blood from the person being tested. At least, this kind of test requires two visits: one to have your blood drawn and the second to receive HIV testing results and medical referrals for HIV care if the results are positive.

**Q65. Of the following testing options, please mark all those that you think you probably would NEVER use**

1. Conventional testing at a HIV/STI specialized clinic/centre

2. Conventional testing at Primary Care

3. Conventional testing al a private laboratory

4. Getting a Rapid test at a General Health Care service (Primary Care/Emergency Department…)

5. Getting a Rapid test at a HIV/STI specialized clinic/centre

6. Getting a Rapid test at a pharmacy by a pharmacist

7. Getting a Rapid test at a Community Based Organization or Non-Governmental Organization

8. Getting a Rapid test at a bar/pub, club or sauna

9. Home self-sampling (you take the sample and then send it to a laboratory who will tell you the result)

10. Self-testing (take the sample, test yourself and interpret your result in under 20 minutes)

11. I would not discard any of them.

**Q66. If all the above mentioned testing options were available in your country: What phrase better describes the way you would use them?**

1. I would only use one kind of testing option

2. I would mainly use one testing option, although I would occasionally use a second one

3. I would use one testing option more often than the others, but I would also use two other types with some regularity.

4. I would use two testing options more or less equally

5. I would mix more than three types of tests.

*[If Q66=1]* **Q67. Please mark which test you would use.**

1. Conventional testing at a HIV/STI specialized clinic/centre

2. Conventional testing at Primary Care

3. Conventional testing al a private laboratory

4. Getting a Rapid test at a General Health Care service (Primary Care/Emergency Department…)

5. Getting a Rapid test at a HIV/STI specialized clinic/centre

6. Getting a Rapid test at a pharmacy by a pharmacist

7. Getting a Rapid test at a Community Based Organization or Non-Governmental Organization

8. Getting a Rapid test at a bar/pub, club or sauna

9. Home self-sampling (you take the sample and then send it to a laboratory who will tell you the result)

10. Self-testing (take the sample, test yourself and interpret your result in under 20 minutes)

*[If Q66=2 OR Q66=5]* **Q68. Please mark which test you would use mainly.**

1. Conventional testing at a HIV/STI specialized clinic/centre

2. Conventional testing at Primary Care

3. Conventional testing al a private laboratory

4. Getting a Rapid test at a General Health Care service (Primary Care/Emergency Department…)

5. Getting a Rapid test at a HIV/STI specialized clinic/centre

6. Getting a Rapid test at a pharmacy by a pharmacist

7. Getting a Rapid test at a Community Based Organization or Non-Governmental Organization

8. Getting a Rapid test at a bar/pub, club or sauna

9. Home self-sampling (you take the sample and then send it to a laboratory who will tell you the result)

10. Self-testing (take the sample, test yourself and interpret your result in under 20 minutes)

*[If Q66=2]* **Q69. Please mark which test you would use from time to time.**

1. Conventional testing at a HIV/STI specialized clinic/centre

2. Conventional testing at Primary Care

3. Conventional testing al a private laboratory

4. Getting a Rapid test at a General Health Care service (Primary Care/Emergency Department…)

5. Getting a Rapid test at a HIV/STI specialized clinic/centre

6. Getting a Rapid test at a pharmacy by a pharmacist

7. Getting a Rapid test at a Community Based Organization or Non-Governmental Organization

8. Getting a Rapid test at a bar/pub, club or sauna

9. Home self-sampling (you take the sample and then send it to a laboratory who will tell you the result)

10. Self-testing (take the sample, test yourself and interpret your result in under 20 minutes)

*[If Q66=3]* **Q70. Please mark which test you would use more often than the others.**

1. Conventional testing at a HIV/STI specialized clinic/centre

2. Conventional testing at Primary Care

3. Conventional testing al a private laboratory

4. Getting a Rapid test at a General Health Care service (Primary Care/Emergency Department…)

5. Getting a Rapid test at a HIV/STI specialized clinic/centre

6. Getting a Rapid test at a pharmacy by a pharmacist

7. Getting a Rapid test at a Community Based Organization or Non-Governmental Organization

8. Getting a Rapid test at a bar/pub, club or sauna

9. Home self-sampling (you take the sample and then send it to a laboratory who will tell you the result)

10. Self-testing (take the sample, test yourself and interpret your result in under 20 minutes)

*[If Q66=3]* **Q71. And which test would you use as the second most frequent?**

1. Conventional testing at a HIV/STI specialized clinic/centre

2. Conventional testing at Primary Care

3. Conventional testing al a private laboratory

4. Getting a Rapid test at a General Health Care service (Primary Care/Emergency Department…)

5. Getting a Rapid test at a HIV/STI specialized clinic/centre

6. Getting a Rapid test at a pharmacy by a pharmacist

7. Getting a Rapid test at a Community Based Organization or Non-Governmental Organization

8. Getting a Rapid test at a bar/pub, club or sauna

9. Home self-sampling (you take the sample and then send it to a laboratory who will tell you the result)

10. Self-testing (take the sample, test yourself and interpret your result in under 20 minutes)

*[If Q66=3]* **Q72. Please mark which test you would use as a third option.**

1. Conventional testing at a HIV/STI specialized clinic/centre

2. Conventional testing at Primary Care

3. Conventional testing al a private laboratory

4. Getting a Rapid test at a General Health Care service (Primary Care/Emergency Department…)

5. Getting a Rapid test at a HIV/STI specialized clinic/centre

6. Getting a Rapid test at a pharmacy by a pharmacist

7. Getting a Rapid test at a Community Based Organization or Non-Governmental Organization

8. Getting a Rapid test at a bar/pub, club or sauna

9. Home self-sampling (you take the sample and then send it to a laboratory who will tell you the result)

10. Self-testing (take the sample, test yourself and interpret your result in under 20 minutes)

*[If Q66=4]* **Q73. Please mark which two tests you would use.**

1. Conventional testing at a HIV/STI specialized clinic/centre

2. Conventional testing at Primary Care

3. Conventional testing al a private laboratory

4. Getting a Rapid test at a General Health Care service (Primary Care/Emergency Department…)

5. Getting a Rapid test at a HIV/STI specialized clinic/centre

6. Getting a Rapid test at a pharmacy by a pharmacist

7. Getting a Rapid test at a Community Based Organization or Non-Governmental Organization

8. Getting a Rapid test at a bar/pub, club or sauna

9. Home self-sampling (you take the sample and then send it to a laboratory who will tell you the result)

10. Self-testing (take the sample, test yourself and interpret your result in under 20 minutes)

**HEALTH AND BEHAVIOURS**

**Remember that, we use "sex" to mean ONLY where there is vaginal or anal penetration**

**With that definition in mind,**

**Q74. How old were you the very first time you had sex with a man?**

[*insert*]

**Q77. In the LAST 12 MONTHS, with how many men have you had sex?**

1. None

2. One

3. 2-4

4. 5-9

5. 10-19

6. 20-50

7. Over 50

**Q78. And with how many did you have an unprotected anal intercourse? (in the last 12 months)**

1. None

2. One

3. 2-4

4. 5-9

5. 10-19

6. 20-50

7. Over 50

**Q75. In the LAST 12 MONTHS, have you given money, drugs or other benefits to a man to have sex with you?**

1. Yes

2. No

**Q76. And in the LAST 12 MONTHS, have you been given money, drugs or other benefits by a man to have sex with him?**

1. Yes

2. No

**Q79. In the LAST 12 MONTHS, in which of the following places have you found someone with whom you have had sex? (Mark all that apply)**

1. Dark Rooms, sex shops

2. Discotheques, clubs and gay bars

3. Saunas

4. Sex Clubs

5. Internet

6. Dating Apps (Grinder, Scruff) 🡪 Go to Q81

7. Parks, public restrooms, and other places for flirting or "cruising"

8. A sex party in a private home

9. Other [Specify]

**Q80. And where did you find the highest number of partners?**

1. Dark Rooms, sex shops

2. Discotheques, clubs and gay bars

3. Saunas

4. Sex Clubs

5. Internet

6. Apps

7. Parks, public restrooms, and other places for flirting or "cruising"

8. A sex party in a private home

9. Other [Specify]

**Q81. Have you ever injected drugs?**

1. Yes, but more than 12 months ago

2. Yes in the last 12 months

3. No, never

*[If Q77≠1]*

**Q82. In the LAST 12 MONTHS, have you taken recreational drugs immediately preceding and/or during sex?**

1. Yes

2. No

*[If Q82=1]***Q83. In these 12 months, which of the following drugs did you take immediately preceding and/or during sex? (Mark all that apply)**

Mephedrone/Methilone (Meow, MCAT, Plant food)

Methamphetamine (Crystal,Tina, Meth, Ice)

Cocaine

Ecstasy/MDMA

Ketamina

GHB/GBL (G, Gina, Liquid ecstasy)

Amphetamine

Poppers

Viagra

Cannabis (Marijuana, Hachis, Sintetic cannabinoids, spice)

Other drugs: Please specify

[If Q83= 1 OR 3 OR 5 OR 6] **Q84.Have you ever injected one or more of the following drugs:**

1. Mephedrone/Methilone (Meow, MCAT, Plant food)
2. Methaphetamine
3. Cocaine
4. Ecstasy/MDMA

**Q85. In these LAST 12 MONTHS, with how many women have you had an unprotected sexual intercourse?**

1. None

2. One

3. 2-4

4. 5-9

5. More than 10

**Q85_2. In the last 12 months, have you been given condoms? (e.g. through an outreach service, drop-in centre or sexual clinic)**
 1. Yes
 2. No

**Q86. Have you ever been diagnosed with any of these sexually transmitted infections? Select ALL that apply**

|  |  |  |
| --- | --- | --- |
| 1. Syphilis |  |  |
| 2. Gonorrhea |  |  |
| 3. Chlamydia |  |  |
| 3. Anal or genital warts |  |  |
| 4. Anal or genital herpes |  |  |
| 1. Hepatitis 2. I haven’t been diagnosed for any of these. |  |  |

*[If Q86>1]* **Q86a. Of those diseases, which was the latest?**

1. Syphilis

2. Gonorrhea

3. Chlamydia

3. Anal or genital warts

4. Anal or genital herpes

5. Hepatitis

*[If Q86a=5]* **Q86b. What type of hepatitis?**

1. Hepatitis A

2. Hepatitis B

3. Hepatitis C

4. Others

**Q87. When was your LAST diagnosis?**

1=Less tan 12 months

2=Between 1 and 2 years

3=Between 2 and 5 years

4=More than 5 years ago

**A LITTLE BIT MORE OF YOU**

**Q89. Which of the following best describes your occupation during the last 12 months?**

1. Employed full-time

2. Employed part-time

3. Self-employed

4. Unemployed (with or without subsidy)

5. Student

6. Retired

7. Long-term sick leave/ medically retired

**Q89_2The current financial situation for your household is:**

1. Comfortable
2. It is all right
3. It is tight, I need to be careful
4. I make ends meet with difficulties
5. I am unable to make ends meet without debt.

**Q89_3 Please choose the types of health insurance (public and/or private) of which you are holder or beneficiary: (Select all that apply)**

1. Public health system (Social security)
2. State based mutuality (for civil servants, armed forces etc.)
3. Private insurance contracted individually or by your company
4. I do not have a health insurance
5. Other situations. Please describe

**Do you disagree or agree with the following statements on a scale of 1 Strongly disagree to 7 Strongly agree? Please do not spend too much time thinking about any one statement.**

**Q90. Obviously effeminate homosexual men make me feel uncomfortable.**

1. Strongly disagree

2. 2

3. 3

4. 4

5. 5

6. 6

7. 7 Strongly agree

8. Does not apply to me

**Q91. I feel comfortable in gay bars.**

[Same response set]

**Q92. Social situations with gay men make me feel uncomfortable.**

[Same response set]

**Q93. I feel comfortable being seen in public with an obviously gay person.**

[Same response set]

**Q94. I feel comfortable discussing homosexuality in a public situation.**

[Same response set]

**Q95. I feel comfortable being a homosexual man.**

[Same response set]

**Q96. Homosexuality is morally acceptable to me.**

[Same response set]

**Q97. Even if I could change my sexual orientation, I wouldn't.**

[Same response set]

**Please say when something last happened, even if this was not typical for you.**

**Q98. When was the last time you were stared at or intimidated because someone knew or presumed you are attracted to men?**

1. Never

2. More than 5 years ago

3. Within the last 5 years

4. Within the last 12 months

5. Within the last 6 months

6. Within the last 4 weeks

**Q99. When was the last time you had verbal insults directed at you, because someone knew or presumed you are attracted to men?**

1. Never

2. More than 5 years ago

3. Within the last 5 years

4. Within the last 12 months

5. Within the last 6 months

6. Within the last 4 weeks

**Q100. When was the last time you were punched, hit, kicked, or beaten because someone knew or presumed you are attracted to men?**

1. Never

2. More than 5 years ago

3. Within the last 5 years

4. Within the last 12 months

5. Within the last 6 months

6. Within the last 4 weeks

**Thank you very much for taking part in this!**

**Please press “submit answers”**

**If you want more information on HIV, please refer to [www.XXXX.SS]**
